# “Breastfeeding in public” for incarcerated women: the baby-friendly steps

**DOI:** 10.1186/s13006-019-0211-3

**Published:** 2019-04-17

**Authors:** Martha Jane Paynter, Erna Snelgrove-Clarke

**Affiliations:** Dalhousie University School of Nursing, 5869 University Avenue, PO Box 15000, Halifax, NS B3H 4R2 Canada

**Keywords:** Prison, Baby friendly, Public, Breastfeeding support, Incarceration. Maternal health, Breastfeeding rate, Women’s health

## Abstract

**Background:**

Women are the fastest-growing population in carceral facilities in Canada. Most incarcerated women are mothers, with above-average parity. The incarceration of women has implications not only for women’s health, but for that of their children. For example, how is breastfeeding and access to human milk supported in the context of imprisonment? Both carceral and health services are publicly-funded and administered in Canada. Due in part to the well-documented ill-health burden of imprisoned women, health and carceral functions overlap in the spaces of confinement. This paper discusses “breastfeeding in public” in relation to imprisoned women: separated from the public, yet in publicly-funded spaces under public servant control. With increasing adoption of Baby Friendly Hospital Initiative (BFI) Ten Steps in Canadian health centres, there is a need to consider the health centre spaces precluded from its application and make visible the women and children affected. This paper uses the BFI Steps as a lens to consider the environment of confinement for the breastfeeding incarcerated person. The exclusion of breastfeeding and access to human milk for imprisoned women and children extends the punitive carceral function beyond the experience of incarceration and beyond the experience of the convicted mother.

**Discussion:**

Carceral facilities lack breastfeeding policies, foundational to breastfeeding support. Despite high fertility and parity among incarcerated women, carceral health care providers are not required to demonstrate maternity and reproductive health care specialization. The overarching mission of carceral institutions remains security, and support for breastfeeding among incarcerated women is hampered in spaces of conflict, punishment, surveillance and control. A minimal requirement to support exclusive breastfeeding is to promote the mother being with the infant and most incarcerated mothers are separated from their infants. Incarcerated women lack support, information, and community connections for extended breastfeeding beyond six months. Carceral facilities are not welcoming environments for breastfeeding families. Despite the incompatibility of breastfeeding with incarceration, BFI Step 10, coordinating discharge, demonstrates opportunity for improvement through community and health care provider engagement.

**Conclusion:**

Incarceration challenges the reach and applicability of the BFI Steps to enhance breastfeeding and to problematize the idea of breastfeeding “in public.”

## Introduction

The rising incarceration of women impacts reproductive health and reproductive experiences, including breastfeeding. Breastfeeding is understood to be the optimal source of nutrition for infants up to 6 months and with complementary foods for two years and beyond [1, 2]. The incarceration of women in the perinatal period challenges the very possibility of initiating breastfeeding. Although the impact on breastfeeding of incarceration is understudied and rates are unknown, recent research in the United Kingdom and the United States demonstrates the imprisoned women have complex feelings towards breastfeeding depending on their experiences and support available [3–6].

The rate of incarceration in Canada is approximately 136 prisoners per 100,000 people [7], far lower than the rate in the United States at 860 prisoners per 100,000 [8]. However, the number of women in federal corrections in Canada is growing, increasing 66% between 2005 and 2014 [9]. Most incarcerated women are mothers and it is estimated 5% of incarcerated women are currently pregnant [10]. The incarceration of women disrupts family formation and reproduction. Although the perinatal outcomes of incarcerated women and their infants have been the subject of several international systematic reviews [11–14], these reviews include few studies that have examined breastfeeding outcomes among incarcerated women.

Research has found women enter prisons with disproportionate physical and mental illness burdens and experience disproportionate infection, injury, and mortality [10]. Incarcerated women face disproportionate burdens of ill-health and histories of traumatic violent abuse compared with non-incarcerated women. Additionally, the sexed and gendered reproductive health experiences of pregnancy, abortion, labour, birth, post-partum recovery, and breastfeeding create an additional layer of health service requirement for incarcerated women when compared to the male incarcerated population.

Due to the health needs of prisoners and especially women prisoners, the punitive function of the public carceral context overlaps with a health service function. In Canada, all federal carceral facilities include health care units staffed by the Correctional Service of Canada (CSC). Provincial jails may provide health care through a provincial or regional health authority, the department of corrections, or an external contractor: in all cases and at both levels, health services are publicly-funded, and publicly administered across the country.

The World Health Organization and UNICEF have called for all facilities that provide maternity and newborn services to adopt the Baby Friendly Hospital Initiative Ten Steps (BFI) to support breastfeeding success [15]. Research demonstrates application of the BFI Steps improves breastfeeding outcomes [16]. In Canada, the rate of breastfeeding initiation is 89% [17]. At last published count, BFI-designated facilities in Canada included twenty-one maternity hospitals [18]. With increasing adoption of BFI Ten Steps in Canadian health centres, there is a need to consider the health centre spaces precluded from its application, such as those who experience criminalization, incarceration, and health services in jails and prisons.

We examine the 2018 revised BFI Ten Steps using a lens that considers the implications for breastfeeding support for incarcerated women. Juxtaposing the conflicts of the carceral space as hidden/public and as punitive/therapeutic, we explore the meaning of breastfeeding in public for incarcerated women. Through literature review and our experience providing support to this population, we examine the public nature of the carceral environment, the demographic and health characteristics of prisoners, the evidence of breastfeeding among the imprisoned population, and carceral policy and infrastructure, to comment on the need for consideration of the BFI Steps in relation to the public institutional space of carceral facilities.

### The carceral space as public

Carceral spaces in Canada are often hidden from the public, located on the outskirts of town, in small towns and rural places. By design, the spaces feature barriers to entry and exit, physical bars, locks, brick walls and electric fencing. While prisoners are hidden from the public, their bodies and activities are under near-constant public surveillance. This surveillance is gendered. For example, federally-incarcerated women have been found to be subjected to the practice of their bodies and cells being searched in a discretionary and unpredictable manner [19]. In the Western world, women prisoners’ health needs are disproportionately interpreted as unruly, or “mad”, and necessitating psychiatric control and observation [20].

In addition to the non-private experience of observation and surveillance, there is the non-private governance and orchestration of incarceration in Canada. Carceral space is definitively public: publicly-funded and administered by the Department of Public Safety at the federal level and by Departments of Corrections at the provincial level. The intention of incarceration is orientated to the public interest: purportedly to protect the public from harm by preventing or deterring crime. Public policy governs operations, and public servants staff the institutions. This “public” existence contrasts with the invisibility of the lived experiences of incarcerated women.

However, prisoners in Canada share protections afforded to the general public, such as equal human rights enjoyed by all persons [21]. The rights include both rights to health and rights to privacy. Several recent high-profile news stories have demonstrated pregnant prisoners are denied the right to equal access to publicly-funded health services [22–25]. As health care facilities across the country adopt BFI practices and receive BFI designation, policy and decision-makers must consider the exclusion of a small but growing and deeply marginalized population of mothers. As breastfeeding promotion advances in public health institutions, will the gap in health experience widen for imprisoned mothers?

### Incarcerated women in Canada

Despite growing numbers, women remain the minority of incarcerated individuals in Canada. There are 692 women imprisoned in the six federal women’s prisons [26], representing 8.4% percent of the total federally-incarcerated population [26]. Women comprise 16% of the 25,000 people admitted to provincial custody annually, approximately 4000 people [27]. Federal sentences are for two years or more, and provincial facilities imprison both individuals with sentences for two years less a day and those remanded to pretrial custody, for which there is no time limit. Sixty percent of incarcerated people in provincial custody are held on remand [27]. Indigenous women and women of colour are over-represented, with over a third of imprisoned women identifying as Indigenous, and over 10% as Black [28].

As a small minority of the incarcerated population, women prisoners face layers of social isolation. For example, women are geographically dispersed to be imprisoned in a small number of women-only facilities or in small units co-located with larger male-dominated prisons. This isolation contributes to federally-incarcerated women experiencing fewer visits [29]. The most immediate health consequence of incarceration is disconnection from social, physical and mental supports such as family, friends, regular primary care providers and community-based therapeutic programs. There is no internet inside carceral facilities for women and limited access to phones, for which there are high usage fees [30]. Prisoner visitors are subject to administrative approval and search, may travel long distances, and are limited in duration of visit and contact.

At the same time as women prisoners experience disproportionate isolation from visitors, prisons for women are increasingly overcrowded inside. Overcrowding reduces privacy and can exacerbate mental health and sanitation concerns. Privacy is a protected right in Canada. Section 7 of the Charter of Human Rights specifies protection of security of the person, and Section 8 stipulates security from unreasonable search [31]. However, “internal reforms have not proven sufficient to bring prison conditions and practices into compliance with the Rule of Law.” [32].

Reformist ideals have resulted in expansion of the carceral infrastructure for women and mothers in Canada in recent years. During the late 1990’s to early 2000’s, five federal prisons and one healing centre for women opened across Canada to replace the Prison for Women in Kingston, Ontario, which closed in May 2000 [33]. In 2001, the CSC implemented the Mother Child Program (MCP), through which children may live full-time, on-site with their mothers in federal carceral facilities [34]. The program applies to every federal facility, including: Nova Institution for Women, Nova Scotia; Edmonton Institution for Women, Alberta; Grand Valley Institute for Women, Ontario; Joliette Institution, Quebec; Fraser Valley Institution, British Columbia; and the Okimaw Ohci Healing Lodge in Saskatchewan. Despite the potential breadth of the program, eligibility criteria for the MCP are restrictive, and use has declined since implementation to a few people per year [34]. In all of Canada, there is one provincial facility with an MCP, the Alouette Correctional Centre for Women in Maple Ridge, British Columbia. Although the program was shut down in 2008, Alouette prisoners launched a successful constitutional challenge and the program reopened in 2016. That decision, *Inglis v BC Minister of Public Safety* [35] asserted the imprisoned women’s constitutional rights to co-reside with their children, however, it did not result in changes in other provincial jails [36].

Inside carceral facilities in Canada, prisoners experience increased risk of injury, illness, mortality, and suicide compared to outside [10, 37]. Despite the risks of the prison environment, proponents of MCP believe keeping mothers and children together prevents psychological, physiological, and developmental harm to the child [38]. Keeping the child and mother together could be supportive of breastfeeding and fundamental to the BFI Steps. Yet the carceral physical space and institutional requirements challenge the possibility of breastfeeding and furthermore, of BFI policy implementation.

### The health of incarcerated women

Incarcerated women experience what can be described as a clear health deficit stemming from histories of trauma, adversity and social determinants of ill-health. In addition to the contextual and structural barriers, these individual identities and experiences contribute to greater risks of not breastfeeding. Among federally-incarcerated women, 70% report having experienced histories of sexual abuse and 86% of physical violence in childhood [39]. While over a third of prisoners are Indigenous, an estimated 15–20% of currently-incarcerated people identify as survivors of Residential Schools [10]. Residential Schools, funded by the Government of Canada and administered by churches, removed Indigenous children from their families and communities; the system undermined Indigenous culture, traumatized families, and included overt abuse [40].

More than half of incarcerated women report physical and mental health needs [10]. Psychotropic medications are more commonly prescribed to inmates than the general population, and more women than men have active prescriptions [41]. Infectious diseases, chronic pain and chronic illness are common. More than half of prisoners are under the age of 35 [27] and most prisoners have not completed high school [28].

### Breastfeeding in the carceral space

The complexity of prisoner health is likely to impact breastfeeding. Psychosocial factors such as stress and lack of support negatively influence breastfeeding duration [42]. Due to trauma histories and lack of exposure to breastfeeding in their upbringing and family life, incarcerated women may feel more unfamiliarity and discomfort with breastfeeding [43]. Separation from children causes emotional harms to incarcerated mothers [44, 45] and custody issues are a critical concern [46]. These concerns are likely to influence the receptivity of incarcerated women to breastfeeding instruction, their intention to breastfeed, and their maintenance of breastfeeding if they are separated from the infant.

Access barriers to research among the women prisoner population generally, and the lack of mother-infant residential units in North American prisons contribute to a lack of research examining breastfeeding for this population. In the United States, which incarcerates the greatest proportion of prisoners in the world, there are approximately nine residential mother-child programs [47] and most incarcerated women will have their children removed from their custody one to two days after birth [6]. Even among examinations of mother-child units and other family-friendly policies in prisons, breastfeeding is rarely examined.

One of the few examples of North American research articles to address the subject of breastfeeding among incarcerated women is a single case study explaining the collaboration required to facilitate getting breastmilk from the imprisoned mother to an infant in the care of its father in the community [3]. Qualitative interviews with 28 incarcerated women in England revealed the need for abundant support to facilitate breastfeeding among incarcerated women, and the significance of Mother-infant co-habitation for women to consider breastfeeding [4]. A qualitative study based in Texas of prisoners who knew their infants would be apprehended within 48 h found only one of twelve participants initiated breastfeeding [45]. A further qualitative study of 20 pregnant prisoners in New York found the uncertainty of imprisonment affected plans for breastfeeding [5]. As interviews were conducted prenatally, no breastfeeding experience was captured [5]. Finally, a recent mixed-methods study in Minnesota is considered the first to provide quantitative results with regards to breastfeeding intention and initiation among imprisoned women [6]. Among 39 participants in a prison-based doula-support program, while less than half indicated an intention to breastfeed at program onset, 69.2% discussed breastfeeding with their doula, and 64.1% initiated breastfeeding after delivery [6]. As these participants were not eligible for mother-baby residential programs, this study reinforces the importance of opportunity and of doula support [48] to breastfeeding initiation for this population. Collectively, this research demonstrates the impact of incarceration on breastfeeding.

To our knowledge and through careful examination of the literature, we have not found any study examining breastfeeding among prisoners in Canada. Neither the provincial jails nor the federal prison in Canada collect data on women’s intentions to breastfeed, initiation of breastfeeding, or duration of breastfeeding. Despite this lack of data, it is possible that the federal MCPcould be supportive of breastfeeding.

## The baby friendly hospital initiative ten steps

### Breastfeeding policy

The first step in BFI is institutional adoption of a breastfeeding policy. In most Canadian provinces, there is but one or two provincial carceral facilities for women. In the East Coast province in which we are situated, there is one facility co-located with a larger men’s jail [49]. This facility does not have a breastfeeding policy, however, there is a breastfeeding policy at the provincial level shared between the health authorities and the provincial department of Public Health [50]. The policy specifies provincial support for breastfeeding to two years and beyond, that all families will be provided evidence-based information about the benefits of breastfeeding and the risks of formula feeding, and that families will experience an environment that is supportive of breastfeeding.

This provincial policy is not operationalized for incarcerated women. Under the custody of a public department (Corrections), the women do not have the privileges of women in public. Not only are infants and children separated from their mothers when their mothers are incarcerated in the provincial facility, these incarcerated women may not make any physical contact with facility visitors, including children, unless specifically ordered by the Court.

At the federal level, the CSC lacks a breastfeeding policy. Federal legislation ensures incarcerated individuals the right to health services. Section 70 of the *Corrections and Conditional Release Act* specifies living and working conditions of inmates must be “healthful” [51]. Section 86 requires inmates receive essential health care and reasonable access to non-essential health care, and Section 87 requires the CSC consider the state of an inmate’s health in all decisions affecting them. Section 77 instructs CSC to provide programs specific to women’s needs. However, the Act fails to attend specifically to breastfeeding or perinatal health. The absence of breastfeeding from the Act could be addressed through policy. At the federal level, policies stemming from legislation are prepared as Commissioner’s Directives (CD). Commissioner’s Directive 800 governs Health Services, and it too does not mention breastfeeding [52].

Commissioner’s Directive 768 governs the Institutional MCP [38]. The purpose of the MCP is “To foster positive relationships between federally incarcerated women and their children by providing a supportive environment that promotes stability and continuity for the mother-child relationship” [38]. Breastfeeding is not mentioned in the Directive. Interestingly, CD-768 Section 64, addressing non-residential components of mother-child programming, states that “institutions are encouraged to implement various non-residential means of establishing and/or maintaining the mother-child bond, including, but not limited to, use of escorted/unescorted temporary absences for family contact/parental responsibilities, private family visits, recording of stories, pumping and storing of breast milk” [38]. While the commodity of breastmilk is acknowledged, the relational experience of breastfeeding is not.

### Training and education to support breastfeeding

The second BFI step requires specific education for health care staff in breastfeeding support. In many provincial carceral facilities in Canada, health care staff are employees of the provincial health authority. In the federal system, health care staff and correctional officers are both employed by CSC. The Office of the Correctional Investigator, an independent watchdog for federal corrections, describes the employment of health care staff under Corrections as presenting clinical and ethical conflicts [53]. These conflicts could include, for example, a health care provider’s knowledge that it is optimal for a client to be able to prepare in advance for a clinical appointment, versus Corrections policies that may disallow any prior communication of an appointment time or date to the client due to security concerns. Another example could be health care providers’ concerns with the use of physical restraints with a client who has a history of trauma, when such restraints are used routinely in Corrections.

Training for breastfeeding support rests with the employers. Despite high fertility among incarcerated women, the carceral health care providers are not specifically required to develop and demonstrate maternity and reproductive health care specialization and skill. Furthermore, the Canadian Nurses Association does not recognize maternity as an area of development for correctional nurses [54].

BFI requires health care staff discuss the importance and management of breastfeeding with pregnant women and their families [15]. Confident, well-informed, compassionate and creative encouragement and support for breastfeeding among incarcerated women is hampered because prisons are spaces of surveillance and control [19]. Breastfeeding is not included in the Guidelines for the Implementation of Mother-Child Units in Canadian Correctional Facilities [55]. While specialized training in breastfeeding for forensic nursing staff may be out of reach, the restrictions on access to external expertise is unduly discriminatory. Despite the complex health needs of this marginalized population and the supplemental needs of women in pregnancy, peripartum and postnatally, incarcerated women are not treated as patients first, but as “offenders” [52]. In the interest of security, prisoners experience limited contact with outsiders, including health professionals and breastfeeding advocates and supports. In the provincial facility for women in our province, prisoners are not permitted to know the dates or times of their external clinical appointments. Families are not invited to participate in these appointments to share in information gathering or in other aspects of perinatal support. Indeed, family contact is deeply limited among incarcerated women.

### Contact

The World Health Organization recognizes that a minimal requirement to support exclusive breastfeeding is to promote the mother being with the infant. BFI Step 4 requires support of immediate and uninterrupted skin-to-skin contact after birth to support breastfeeding initiation [15]. This is beyond the scope of responsibility of carceral facilities, as none in Canada provide intrapartum care through in-house health services. At the federal level, Section 20 of CD-800 states that “For pregnant offenders, Health Services will ensure arrangements for childbirth are made at an outside hospital” [52]. During an inpatient admission, the outside hospital policies and practices can influence support for breastfeeding. In advance of this outside support, the value of the “golden hour” [56] should be communicated to incarcerated women and be supported by correctional officers present at birth. There is no data currently available to inform understanding of the extent to which this occurs. As incarcerated women have two correctional officers present in their postpartum room until discharge, they lack privacy from correctional officers to place their newborns skin-to-skin.

Shackling and use of restraints would physically, and emotionally, impair the golden hour and skin-to-skin contact. In the United States, over twenty states have enacted anti-shackling legislation [57], there is no legislation in Canada that specifically bans the shackling of prisoners. Furthermore, the lack of evidence-based, gender-sensitive, breastfeeding-promoting policy in carceral health services administration conflicts with public health efforts and messages for the early postpartum period.

Baby Friendly Hospital Initiative Step 7 requires mothers and children room together [15]. Because of the high rates of substance use disorder among criminalized women, **i**ncreasing adoption of rooming-in and maternal therapy as the first line intervention in treating neonatal abstinence syndrome [58] has significant implications for redressing carceral norms that would otherwise result in separation of mother and infant. Notably, for federally incarcerated women, CSC must pay the provincial health authorities for costs of care, including extended stays in hospital required for maternal therapy for neonatal abstinence syndrome.

Step 5 requires staff support mothers to maintain breastfeeding and manage common difficulties [15]. In addition to physical separation from their infants, incarceration presents emotional challenges to breastfeeding success. Histories of abuse, their own placement in foster care as children, and other socio-economic factors place incarcerated women at high risk of experiencing attachment disorders [59, 60]. Coercive practices such as strip searching, administrative segregation (solitary confinement), and restraints can trigger emotional distress [61]. The confining experience of incarceration may cause women to experience increased anxiety and depression [61]. Demonstration of sensitivity to these additional challenges by correctional and health service staff may support incarcerated women’s emotional health in the postpartum period [62] and facilitate breastfeeding.

While the federal residential MCP would allow for infants and mothers to reside together to facilitate exclusive breastfeeding, few qualify [34, 63]. Most incarcerated women are in provincial, not federal, facilities [7]. At the time of federal implementation of the MCP in 2001, there were 375 women in federal prison and twelve participants [34]. Over the next ten years, the federally-incarcerated population doubled, while MCP participation dwindled to no more than three participants a year [34]. Use of the MCP varies by institution [34]. In 58% of the months from 2001 to 2012, the Joliette Institution had full-time participants, making it the most active program [34]. The Edmonton Institution for Women never had a participant and Okimaw Ohci has not had a participant since 2005 [34]. Data regarding MCP participation from 2012 to the present is not publicly available. However, it is known that in 2014, CSC added 114 minimum security beds to its facilities for women, and 15 new rooms specifically for mother-child pairs [64]. Prioritizing the health benefits of breastfeeding for both mother and infant could support expansion of MCP participation federally and of MCP programs in provincial facilities.

### Support exclusive breastfeeding

BFI Steps 6, 8 and 9 relate to provision of informed support for exclusive breastfeeding [15]. For example, in Step 6, staff are urged to refrain from providing newborns any food or fluids other than breast milk, unless medically indicated. Although not included in the World Health Organization’s acceptable reasons for breast milk substitute supplementation, “medically indicated” may be interpreted to include social indications, such as separation from the mother [65]. Step 9 includes counseling mothers on the use and risks of feeding bottles, teats and pacifiers [15]. While prisoners need up-to-date clinical information and support about the risks of artificial teats, when mothers are unable to be present due to incarceration, artificial teats and pacifiers may become necessary.

When faced with separation, contact visits and the assurance that infants will receive pumped milk may improve breastfeeding intention, initiation and duration among incarcerated women. However, carceral spaces are not clean or convenient sites for breastmilk pumping and storage. Prisoners are surveyed, subject to search, and must respond to head count and institutional schedules and requirements. Even if equipment, supplies and storage to facilitate milk expression were made available, the psychological toll of incarceration is likely to impede success.

Step 8 involves supporting mothers to recognize and respond to their infants’ cues for feeding [15]. Even within the MCP program, carceral institutions enforce schedules and strictly structure prisoner time. This is a challenging context for teaching and learning cue-based, responsive feeding. Mother-infant dyad participants in the MCP are rarely separated; on occasion another prisoner may qualify as a babysitter for brief periods [38]. While this continuous contact can be emotionally and physically taxing for mothers inside, it may support extensive observation of their infants and development of understanding of their cues if education is available [66].

Unlike some other jurisdictions with short-term mother-child programs, the federal MCP in Canada extends to six years of age. In the West, most prison nurseries and Mother-Baby Units allow children to stay only up to a maximum age of 18 months [47]. The longer-term model in Canada is conducive to extended breastfeeding [38]. However, incarcerated women participants in MCP lack role models, peer support, information, and community connections. The potential for greater emphasis on breastfeeding to create additional pressure and feelings of poor self-esteem among incarcerated women, particularly given the contextual and structural restrictions on feeding infants at the breast, must be considered. The positive prisoner response to breastfeeding demonstrated in the existing research points to the great potential for breastfeeding education and support to empower this population [5, 6].

### Coordination

BFI Step 10 stipulates that health facilities coordinate discharge so that parents and their infants have timely access to ongoing support and care [15]. As we have described, carceral facilities are geographically isolated and women are often displaced far from their homes, support networks and families. Prisoners have limited contact with public health resources in nearby communities, and support people face significant barriers to institutional entry to provide support on site.

Despite the incompatibility of breastfeeding with the carceral function and the carceral space, this final step demonstrates the most space for immediate opportunity. Step 10 can be achieved through building relationships between corrections and external health care providers and peer support with expertise and experience in lactation, including public health nurses, community midwives, lactation consultants, doulas, lay supports and volunteers. For incarcerated women, access to outside appointments/programs depends on approval for temporary absences and, in the federal system, on cleared persons to escort [67]. There are two immediate potential areas of improvement: 1) developing ease of application for temporary absence permits; and 2) improving volunteer escort rosters to facilitate access to these programs in the community. These interventions could facilitate access to breastfeeding education, support and care. In the longer term, considerations of the importance of breastfeeding can inform the development of alternatives to incarceration for pregnant and postpartum women, and shift norms such that incarceration is used not with increasing frequency, but rather as a last resort.

## Conclusion

In this paper we describe the public nature of the under-considered environment of carceral facilities for breastfeeding research, policy and practice. We demonstrate the incompatibility between advocacy for public health environments and services that are supportive of breastfeeding, as outlined in the BFI Ten Steps [15], and the escalating incarceration of high-health-needs women and mothers in federal and provincial facilities. The infrastructure, policies and practices of incarceration impinge upon breastfeeding. Limited evidence suggests that with education and support, incarcerated women may overcome the barriers of the carceral space to initiate breastfeeding [6]. There is no research to demonstrate what interventions promote breastfeeding maintenance for this population.

In promotion of breastfeeding in public, we must be conscious of who is missing from public view, and yet under public custody: incarcerated women. Public services that adopt breastfeeding policies, such as health authorities, must be accountable to the women their policies leave out. The decision to adopt and promote BFI must not only consider institutional hurdles in hospitals and community health services, but who is excluded from these environments, and how they might be reached.

## Abbreviations

BFI: Baby Friendly Hospital Initiative
CD: Commissioners Directive
CSC: Corrections Service of Canada
MCP: Mother Child Program
